# Bioactive Compounds, Food Fermentation, and Sustainable Valorization: Advances, Gaps, and Future Directions

**DOI:** 10.3390/molecules31122115

**Published:** 2026-06-16

**Authors:** Irene Dini

**Affiliations:** Department of Pharmacy, University of Naples Federico II, 80131 Napoli, Italy; irdini@unina.it

The growing global emphasis on sustainability, public health, and food innovation is reshaping the scientific and industrial perceptions of plant-based bioactive compounds and agrofood by-products [1]. Once considered low-value residues, materials such as broccoli stalks [2], artichoke waste [3], mango leaves [4], olive pomace [5], grains [6], and non-commercial fruits [7] are now recognized as rich reservoirs of functional molecules with nutritional, technological, and health-promoting potential. The contributions collected in the Special Issue “Bioactive Compounds, Food Fermentation, and Sustainable Valorization: Advances, Gaps, and Future Directions” illustrate how these matrices can be transformed into high-value ingredients, supporting a circular bioeconomy while addressing modern dietary and health challenges. Plant by-products such as dietary fiber [8], polyphenols [9], flavonoids [10], phenolic acids [11], glucosinolates [12], carotenoids [13], and saponin [14] can be concentrated or structurally modified through innovative processing techniques, enhancing their functional properties and bioavailability [15]. Margarit et al. demonstrated that broccoli stalk powder obtained by freeze-drying can be incorporated into bread formulations, significantly increasing total dietary fiber, polyphenol content, and antioxidant activity [contribution 1]. Quizhpe et al. explored the use of enzymatically hydrolyzed artichoke and broccoli residues to fortify gluten-free breads, a category often criticized for poor nutritional quality. Their findings show substantial increases in soluble dietary fiber, phenolic compounds, and antioxidant capacity, accompanied by improved starch digestibility and reduced predicted glycemic index [contribution 2]. Lawrence et al. conducted a comprehensive prescreening of mango leaves from different cultivars, revealing significant variability in techno-functional properties and antioxidant profiles. Rich in mangiferin, quercetin, and kaempferol, mango leaves emerge as promising natural ingredients for functional foods and nutraceuticals [contribution 3]. Ziobro et al. evaluated the incorporation of a by-product mixture composed of apple pomace, red potato pulp, and sugar beet pulp (1:1:1) into corn-based extruded snacks. Their findings show that a 20% inclusion level provides the most balanced technological and nutritional outcome. Specifically, the formulation enhances dietary fiber, polyphenols, anthocyanins, tocopherols—particularly α-tocopherol—and key phytosterols (β-sitosterol, stigmasterol, campesterol), while reducing starch content and maintaining acceptable physical quality. This study further supports the concept that agro-industrial residues can be transformed into functional ingredients capable of improving both the health value and sustainability profile of widely consumed snack products [contribution 4]. Across these studies, two technological strategies stand out: enzymatic biotransformation, which releases bound phenolics and increases soluble fiber fractions, and freeze-drying, which preserves thermolabile compounds and enhances extractability. Together, these approaches demonstrate how processing can convert agricultural residues into potent functional ingredients. A second major theme concerns the biological activities of plant extracts and their potential applications in preventing or mitigating chronic diseases. Several contributions provide mechanistic insights into how bioactive compounds modulate oxidative stress, inflammation, metabolic pathways, and neuronal health. Gavia-García et al. reported that supplementation with *Sechium edule* var. *nigrum spinosum* in older adults with type 2 diabetes mellitus significantly increased SIRT1, SIRT3, SIRT5, and SIRT6 expression while improving systemic antioxidant status. These findings suggest a role for chayote in modulating mitochondrial function, metabolic homeostasis, and oxidative stress, key factors in diabetes management [contribution 5]. Bouaita et al. examined the influence of solvent polarity on the phytochemical profile and bioactivity of *Elaeagnus angustifolia* leaf extracts, demonstrating strong antioxidant activity and α-amylase inhibition, particularly in hydroethanolic fractions. These results support the plant’s traditional use and highlight its potential as a natural antidiabetic agent [contribution 6]. Boutora et al. characterized the phytochemical composition of *Ajuga iva* and demonstrated its anti-inflammatory, analgesic, and antipyretic activities, supported by molecular docking analyses showing strong interactions with COX-2 [contribution 7]. This integrative approach reinforces the therapeutic relevance of traditional medicinal plants. You et al. investigated hydroxytyrosol-enriched olive pomace juice and showed that it activates the Nrf2, AMPK, SIRT1, PGC1α, and CREB/BDNF pathways, thereby protecting neuronal and microglial cells from oxidative stress and inflammation [contribution 8]. This study exemplifies how olive oil by-products can be repurposed into neuroprotective ingredients. Lee et al. standardized germinated oat extracts rich in avenanthramides and demonstrated their ability to reduce Aβ1-42-induced cytotoxicity and reactive oxygen species in SH-SY5Y cells [contribution 9]. These findings highlight the potential of germinated grains for preventing neurodegenerative diseases. Across these studies, a common mechanistic framework emerges: a reduction in oxidative stress (Nrf2 activation, ROS scavenging), modulation of inflammation (COX-2 inhibition, cytokine reduction), improvements in metabolic regulation (SIRT activation, α-amylase inhibition), and neuroprotection (BDNF upregulation, mitochondrial support). This convergence underscores the relevance of plant bioactives in addressing diabetes, neurodegeneration, and chronic inflammatory conditions. Several contributions demonstrate how plant extracts and by-products can be incorporated into food matrices to improve nutritional quality, sensory attributes, and functional properties. Incorporating broccoli stalk powder into wheat bread not only enhances fiber and antioxidant content but also aligns with consumer demand for sustainable, plant-forward products [contribution 1]. This approach exemplifies how bakery products can serve as vehicles for bioactive delivery. The enzymatically treated artichoke and broccoli extracts used by Quizhpe et al. significantly improved the nutritional profile of gluten-free breads, increasing soluble fiber and reducing glycemic response [contribution 2]. These findings are particularly relevant for individuals with celiac disease or gluten sensitivity, who often face limited access to nutritionally balanced products. Satora et al. investigated cloudy fruit juices fermented with *Lactiplantibacillus plantarum* and lactic acid-producing *Lachancea* yeasts, observing increases in organic acids, polyphenols, and fruity aromatic compounds. These beverages exhibit potential probiotic properties and represent a promising category of functional drinks [contribution 10]. Fermentation emerges as a key technology for increasing bioactive compound content, improving sensory profiles, generating new metabolites with health benefits, and stabilizing functional ingredients. A comprehensive review by Rymuszka and Gorczynska provides a modern reinterpretation of classical fermentations, including alcoholic, acetic, butyric, lactic, and propionic fermentation, highlighting their relevance in contemporary biotechnology [contribution 11]. Dini provided an extensive review of fermented foods, probiotics, and postbiotics, emphasizing microbial metabolism, safety considerations, and the generation of bioactive metabolites such as bacteriocins, exopolysaccharides, and hydroxylated fatty acids [contribution 12]. The review highlights the need for standardized microbial characterization, improved regulatory frameworks, and deeper mechanistic understanding of strain-specific effects. Finally, studies by Birichi et al. [contribution 13], Sakuraoka et al. [contribution 14], and Bouaita et al. [contribution 6] reinforce the growing recognition that plant biodiversity, agricultural by-products, and staple crops represent a vast and largely untapped reservoir of functional molecules. Birinci and colleagues conducted a comprehensive evaluation of *Prunus armeniaca* cultivars, demonstrating significant variability in phenolic composition, antioxidant activity, and functional potential. Their findings highlight the importance of cultivar selection in the design of functional foods and underscore the value of fruit biodiversity as a source of bioactive compounds. This paper complements the studies in the Special Issue by reinforcing the concept that non-commercial or underutilized fruit varieties can serve as high-value functional ingredients [contribution 13]. Sakuraoka et al. identified and characterized active anti-inflammatory compounds in sweet potato roots, demonstrating their ability to modulate inflammatory pathways. This study provides compelling evidence that staple crops, often consumed for their macronutrient content, also harbor potent bioactive molecules with therapeutic potential [contribution 14]. Bouaita et al. (2026) provide a concise yet valuable contribution to the growing body of literature on *Elaeagnus angustifolia*, a species traditionally used in folk medicine across Central Asia and the Mediterranean. The study highlights the rich phytochemical profile of the leaves, emphasizing the presence of phenolic acids, flavonoids, and other antioxidant constituents that collectively underpin the plant’s biological potential. The authors report marked antioxidant activity, which aligns with the high phenolic content and supports the traditional use of *Elaeagnus angustifolia* in managing inflammatory and oxidative stress-related conditions. Additionally, the study documents moderate antimicrobial and enzyme-modulating effects, suggesting possible applications in functional foods or nutraceutical formulations [contribution 6]. 

De Souza Aquino et al. (2026) highlight the functional potential of acerola fruit by-products within sustainable valorization strategies, showing that pulp and processing residues, particularly pomace, peels, and seeds, are enriched in ascorbic acid, hydroxycinnamic acids, flavonoids, carotenoids, and bioactive polysaccharides, often at levels exceeding those found in the edible fraction of food [contribution 15]. The contributions in this Special Issue demonstrate that plant by-products are valuable bioactive resources, not waste materials; processing technologies, including enzymatic hydrolysis, germination, freeze-drying, and fermentation, maximize the release and bioavailability of functional compounds; plant extracts can modulate key biological pathways (Nrf2, SIRT, AMPK, COX-2, NF-κB), supporting antioxidant, anti-inflammatory, antidiabetic, and neuroprotective effects; their incorporation into traditional foods (bread, gluten-free products, fermented beverages) enables the development of sustainable, health-promoting products, and classical fermentations can serve as advanced biotechnological platforms for producing functional ingredients and bio-based chemicals provided that selected microorganisms are used whose potential toxicity is known. Together, these studies offer a multidisciplinary, forward-looking perspective on the future of functional foods, in which sustainability, scientific innovation, and human health converge to transform plant matrices and agro-food by-products into powerful tools for nutrition and well-being.
